# Parents’ experiences of their adolescent child’s depression: a qualitative systematic review and meta-synthesis

**DOI:** 10.1186/s40359-025-03923-2

**Published:** 2026-01-09

**Authors:** Natalia Kika, Jeffrey Lambert, Nina Higson-Sweeney, Vuokko Wallace, Hebah Bhatt, Grace Perry, Shirley Reynolds, Maria Loades

**Affiliations:** 1 Department of Psychology, University of Bath, 10 West, Bath, BA2 7AY United Kingdom; 2 Department for Health, University of Bath, 1 West, BA2 7AY Bath, United Kingdom; 3 Department of Experimental Psychology , University of Oxford, Life and Mind Building, South Parks Road, Oxford, OX1 3EL United Kingdom; 4 School of Psychology and Clinical Language Sciences, University of Reading, Reading, RG6 6UR United Kingdom

**Keywords:** Adolescents, Mental health, Parents, Depression, Help-seeking, Meta-synthesis, Qualitative

## Abstract

A third of adolescents aged 11 to 19 years report depression symptoms, yet many go undiagnosed or do not receive timely treatment. Those who do seek support often face barriers including stigma and long waiting times, resulting in a significant needs-access gap for adolescents looking to access treatment for depression. Parents play a central role in recognizing adolescents’ symptoms and seeking treatment for them. To deepen the understanding on how to better support parents to support their adolescent child, this meta-synthesis aimed to systematically review qualitative studies on parents’ lived experiences of having an adolescent with depression. A pre-planned systematic search using five databases identified 25 papers meeting full inclusion criteria. Data were extracted and a thematic synthesis was conducted using NVivo, with reporting following PRISMA guidelines. Six themes were generated: (1) How do you know when your adolescent has depression?; (2) Understanding the causes of adolescent depression; (3) Emotional turbulence in parents; (4) Effects on the whole family; (5) Experiences with help-seeking; and (6) Stigma and judgment from others. The findings collectively highlight the need for increased parental involvement in professional treatment provision for adolescent depression, alongside better support for parents’ own wellbeing, and improved access to psychoeducation and parent-directed interventions for adolescents with depression.

**PROSPERO registration ID** CRD42024527144.

**Supplementary Information:**

The online version contains supplementary material available at 10.1186/s40359-025-03923-2.

## Highlights

This systematic review synthesizes evidence on the lived experience of parents of adolescents with depression.

25 papers published between 2004 and 2025 were included in the meta-synthesis.

Parents struggle to recognize depression symptoms in adolescents and report self-blame, helplessness and distress.

Parents are motivated to initiate the help-seeking process, but their experiences with professional support vary.

There is a need for parent-directed support and increased parental involvement in adolescent depression treatment.

**Supplementary Information:**

The online version contains supplementary material available at 10.1186/s40359-025-03923-2.

## Introduction

Depression is common in adolescence, with 34% of those aged between 11 and 19 years reporting symptoms, including feelings of helplessness, sadness, hopelessness, and irritability [1]. An increase in adolescent depressive symptoms was found globally during the COVID-19 pandemic, with concerns growing that post-pandemic rates will remain high with long-term consequences [2–4]. Despite the high prevalence, symptoms of adolescent depression often go undiagnosed and untreated, which can lead to an increased risk for subsequent depression and anxiety in adulthood [5]. Even when adolescents do recognize that they are struggling with symptoms of depression, there are barriers to accessing timely and effective support, including stigma, proximity and time constraints [6, 7]. Those who do access Child and Adolescent Mental Health Services (CAMHS) often face extended waiting times due to a lack of capacity or may not be accepted for treatment [8], meaning that only adolescents with severe symptoms may meet the criteria to access help [9]. Therefore, there is a significant needs-access gap in receiving early help for depression symptoms in adolescents, prompting the need for alternative avenues of support including from those who are most present in adolescents’ daily lives, such as parents and caregivers [10, 11].

Parents and caregivers (henceforth collectively referred to as “parents”) commonly initiate help-seeking for adolescent depression. Parents can help by recognizing early symptoms, reaching out for professional support, or supporting their adolescent to do this themselves. Studies have shown that when parents do not recognize symptoms, adolescents are less likely to receive support from mental health services [12, 13]. Primary care professionals also agree that parents play a crucial role in their adolescent accessing and adhering to treatment for depression symptoms [14]. A school-based survey of 23,927 adolescents aged 11–18 years in England found that informal support networks are most commonly sought among the total sample and adolescents with elevated depression and anxiety symptoms (23.1% and 36.2%, respectively). 14% of the total adolescent sample and 21.0% of those with elevated symptoms reported reaching out to their parents for support, highlighting that parents are a commonly sought source of support for adolescents [15]. While their role in help-seeking for adolescents has been established, a systematic review found that parents report barriers to accessing professional support for their adolescent [16]. These included systematic and structural issues, parental views and attitudes towards services and treatment, parental knowledge and understanding of mental health problems and the help-seeking process, and family circumstances [16].

Parents’ involvement in recognizing depression in their child, and then seeking help for them, has theoretical underpinnings through help-seeking models such as the one by Rickwood and Thomas [17]. The model states that problem recognition is a key predecessor to the help-seeking process, which could explain why the lack of parental awareness can delay help-seeking in adolescence. In addition to facilitating help-seeking, parents may also be able to directly support their adolescent to cope with depression symptoms. Evidence from a national survey including 982 parents of young people in Australia found that parents want to, but do not always have the knowledge and resources to do so [18]. Indeed, a systematic review of qualitative and quantitative studies on parental mental health literacy for adolescents found that parents’ knowledge is limited [19]. Higher mental health literacy levels were found among parents who had previous experience of supporting a child with a mental health problem, and variations in mental health knowledge across cultures were also present [19]. Therefore, it is clear that many parents experience uncertainties over when and how to seek support, particularly those facing such challenges for the first time. Meanwhile, there is currently no structured guidance on how parents should be involved in the treatment of adolescent depression [6].

Despite the evidence regarding parents’ key role in adolescent help-seeking for depression, little is known about their lived experiences and what their needs are for support. While individual qualitative studies on the topic have been conducted to explore parental experiences, attitudes and beliefs, these have not yet been synthesized, posing a challenge to making recommendations for addressing parents’ needs and concerns. Conducting a meta-synthesis of qualitative studies could address this challenge by providing insight into parents’ lived experiences across studies, which could expand our understanding of adolescent depression through the lens of their parents. Collated qualitative evidence on parents’ experiences would also complement the available meta-syntheses of adolescent’ lived experience with depression [20, 21] which is crucial to complete the picture on the lived experience not only of adolescents themselves, but also of those involved in their daily lives and support seeking. Therefore, the aim of this systematic review is to synthesize existing qualitative studies to explore the experiences of parents whose adolescent child is struggling with depression.

## Methods

The systematic review protocol was pre-registered on PROSPERO (registration number: CRD42024527144). The review was guided by Enhancing transparency in reporting the synthesis of qualitative research (ENTREQ) guidelines [22] and is reported in accordance with Preferred Reporting Items for Systematic reviews and Meta-Analyses (PRISMA) guidelines [23].

### Inclusion criteria

The inclusion criteria were developed to select studies including in-depth qualitative data reporting on parents’ experiences with their adolescents’ depression symptoms, shown in Table 1. Adolescents who had depression symptoms could be identified by their own self report, by parent and/or teacher report, or by a professional/clinical diagnosis. This broad definition of depression was selected to include a wide range of studies and to capture the experiences of parents whose adolescents may be at different stages of depression.

**Table 1:** Inclusion and exclusion criteria for selection of studies

	Inclusion	Exclusion
Participants	• Parents or primary caregivers of adolescents aged 10–19 (or where the mean age of the adolescent sample falls within this range)	• Parents of children/adults falling outside of the age range of 10–19 (or the mean age is outside of the range)
Type of study	• Empirical studies reporting on parents’ experiences in relation to their adolescent’s depression symptoms • Qualitative or mixed methods • Studies reporting on primary qualitative data involving first person accounts through, interviews, focus groups etc. • Published in a peer-reviewed journal • Written in English, Italian or Spanish • No restrictions on date of upload	• Studies focusing on parents’ experiences in relation to other mental or physical health conditions • Studies reporting secondary data

### Search strategy

The search strategy was pre-planned using the SPIDER tool [24] to identify search terms relating to parents/carers, adolescents, depression, and qualitative studies, shown in Table 2. The full search strategies for each database are available in Additional File 3. The strategy was refined in consultation with a specialist librarian after which five databases were searched in April 2024: MEDLINE, Web of Science Core Collection, PsycINFO, CINAHL and EMBASE. These databases were chosen because they contain peer-reviewed literature on mental health, psychology, social and behavioral sciences. A combination of keywords and MeSH terms was used. No date limits were applied since this is the first review on the subject. Only peer reviewed studies were included, and reference lists of included full-text papers were hand searched for relevant papers.

**Table 2:** Search strategy

Concept	Search Terms
Sample	(parent* or mother or father or caregiver* or guardian* or paternal or maternal or carer*) AND (adolescen* or teen* or young person or young people or youth or student*)
Phenomenon of Interest	depress* or low mood
Design and Evaluation	experience* or need* or attitude* or help*seek* or knowledg* or information or qualitative or interview* or focus group
Research type	qualitative* or qualitative study

### Study selection

The original searches were run in April 2024, and updated searches were conducted in May 2025 using the same databases and search terms to ensure that any additional publications since the initial searches were captured. Database search results were imported into Covidence where duplicates were removed. In the initial stage of screening, paper titles and abstracts were reviewed against the eligibility criteria. Subsequently, full-text screening was conducted for the papers meeting eligibility criteria in the initial stage, and when their eligibility was uncertain. Titles and abstracts were screened by multiple pairings of reviewers from the authorship team and a trained research apprentice who was a psychology student, with 90% average agreement. In the updated search, NK reviewed all abstracts and titles, 30% of which were double-screened by NH-S with 95% agreement. All full-texts were reviewed by NK, and 30% were double-screened with 80% average agreement; NH-S double screened 30% of the full-text papers in the updated search with 100% agreement. Any conflicts between reviewers were discussed between them with reference to the review protocol, and ML was consulted if an agreement was not reached.

### Quality assessment

The Critical Appraisal Skills Programme tool [25] for qualitative studies was used to assess the methodological quality of each study included. This tool was chosen because it is user-friendly and the most commonly used structured framework designed to specifically evaluate qualitative studies [26]. For mixed methods studies, only qualitative findings were evaluated. Each criterion on the 10-item checklist was rated as either ‘yes’, ‘no’, or ‘can’t tell’ to assess whether each included study had a clear statement of aims; used an appropriate methodology; had an appropriate research design and recruitment strategy; considered data collection, research relationships and ethical issues; analyzed data rigorously; stated findings clearly; and added value to the wider literature. All studies were evaluated by NK, with a further 30% inter-rated by HB with 80% agreement in the initial searches, and 30% inter-rated by NH-S with 90% agreement in the updated search. Where ‘can’t tell’ or a disagreement was indicated, ML was consulted to reach a decision. Studies with lower methodological quality were not excluded since this is the first review in this area but their limitations were considered when interpretating the results.

### Data extraction and meta-synthesis

The method of data analysis was a meta-synthesis following the steps outlined by Lachal et al. [27] and guidance from Thomas and Harden [28]. Following data extraction and quality appraisal, the analytical steps involved reading and re-reading each study, line-by-line coding of qualitative results sections in the studies including direct participant quotes from participants, and authors’ reporting of the findings. These were grouped and categorized into themes across studies, after which analytical meta-themes were developed. The authors adopted a critical realist, contextualist ontological and epistemological stance. The critical realist approach posits that there is reality is fallible and therefore no single, objective reality can be obtained from participants [29]. Integrating it with a contextualist stance acknowledges that participants’ knowledge of reality is a result of the unique contexts they are situated in [29].

The data extraction and meta-synthesis process was led by NK, with 30% also extracted by a psychology undergraduate apprentice. Extraction disagreements were discussed and resolved with the authorship team. First, a data extraction spreadsheet was created in Excel according to the guidance by Lachal et al. [27]. Data included were the authors, country, study setting and recruitment method, population and sample including the age range of the adolescents and parents where available, the qualitative data collection method and data analysis approach.

Following data familiarization by reading and re-reading eligible papers, relevant findings were selected from individual papers, including primary data findings and direct quotes. In studies which also included non-parent participants, only findings explicitly linked to parents were coded. Relevant sections were then imported into NVivo 14 software [30] where line-by-line coding of each study was conducted. The codes were then grouped and categorized into descriptive themes and sub-themes based on similarity in meaning, using an inductive data-driven approach due to the exploratory nature of the research question (Additional File 4). The final step involved the development of analytical meta-themes with the study team to create a higher level of interpretation which synthesized relationships and overarching concepts between the descriptive themes across studies.

### Public and patient involvement

A member of the study team’s Parent Advisory Group was consulted on two themes during the meta-synthesis process to seek their input on the interpretation of findings relating to parental recognition of symptoms, and changes to the whole family in terms of adolescent depression.

### Reflexivity

Reflexivity, referring to the process of the authors actively reflecting on how their own experiences, biases and views may impact the interpretation of study findings, was a core component of this meta-synthesis, and predominantly engaged with through reflexive group discussions and the lead author’s reflexivity log. As a team of early-career (NK, NH-S, HB, GP), mid-career (JL, VW) and senior researchers (SR, ML) working in the field of adolescent depression, we approached this systematic review with preconceived attitudes, knowledge and experiences in this area, which drove the focus of this review. Additionally, some of the authors are clinical psychologists (VW, SR, ML), and have current or previous experience of undertaking clinical work with parents and adolescents with depression. These authors were mindful of how their clinical experiences could have led to expectations from the extracted data, and a focus on themes which address challenges they have seen in practice, such as seeking timely support. Furthermore, some of the authors are parents (JL, VW, SR, ML) whereas others are not (NK, NH-S, HB, GP), meaning there were different degrees of relatability to the data, which could have influenced the focus. Finally, NK is conducting a PhD to develop an online intervention for parents of adolescents with low mood, which will be informed by these findings; as such, she closely reflected on how this end goal could influence her interpretation of the data.

## Results

### Study selection and characteristics

**Fig. 1:**
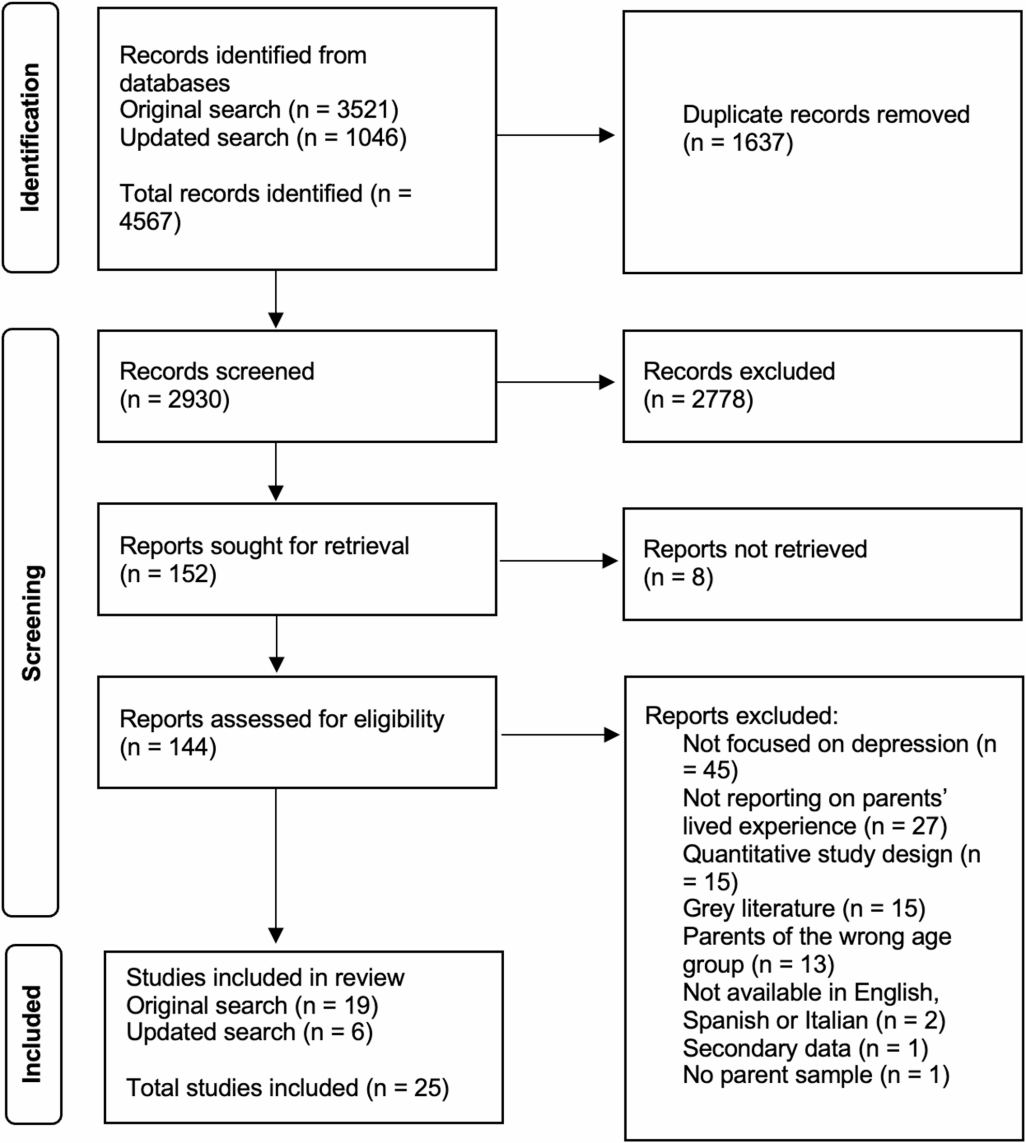
PRISMA flowchart [23] of study selection process from the original (April 2024) and updated searches (May 2025). Note. Of the total numbers, in the updated searches (May 2025) *n* = 139 duplicates were removed; *n* = 907 abstracts and titles were screened; *n* = 23 full texts were screened

The original searches resulted in 3,521 papers, with an additional 1,046 papers identified in the updated searches. Following duplicate removal, the titles and abstracts of a total of 2,931 unique papers were screened for eligibility. At the next stage, 152 full texts were screened and 25 met eligibility criteria to be included in the review (see Fig. 1).

 Characteristics of the included studies are presented in Table 3. A total of 523 parents of adolescents were included in 25 eligible studies. Sample sizes ranged from five to 60. Only seven studies reported on parental gender, and in six of those studies mothers formed most of the parent sample. 40% of the studies (*n* = 10) were conducted in Europe of which seven were in the UK, seven in Asia, three in South America, three in North America, and two in Africa. Parental age was reported in nine studies and ranged from 27 to 64; child age was reported across all studies and ranged from six to 19. Most studies (*n* = 18) recruited parents of adolescents who were receiving professional mental health support from inpatient or outpatient clinics. The majority used a qualitative design (*n* = 22), with three employing a mixed-methods approach. The studies predominantly used semi-structured interviews to collect data (*n* = 23), with two studies using focus group discussions. The most common analytic approach was thematic analysis (*n* = 18). Fourteen studies included parent-only samples; the remaining 11 also explored the experiences of adolescents, teachers, policy makers, or healthcare professionals. Three studies explored parents’ experiences with other mental health conditions in addition to depression, one explored ADHD and autism in addition to depression, respectively, and one explored depression in adolescents with HIV.

**Table 3:** Characteristics of included studies

Author (year)	Country	Participant group; parent sample size, parent gender (where data provided)	Setting	Child age range (Mean age where data provided)	Parental age range (Mean age where data provided)	Qualitative data collection method	Analysis approach
Armitage et al. (2020) [31]	United Kingdom	Parents; *N* = 8	Public mental health service for children and adolescents in the south of England	13–18	N/a	Semi-structured interviews	Interpretative phenomenological analysis
Ckukwuere et al. (2022) [32]	South Africa	Parents; *N* = 14	Mental health units in the North West province	14–17	N/a	Semi-structured interviews	Thematic analysis
De Silva et al. (2020) [33]	United States	Parents; *N* = 15	Self-identified Latinx or Hispanic parents who had concerns about their child’s worry and/or sadness - low-income urban neighborhoods in a large city on the west coast.	6–13 (10.53)	N/a (40.47)	Semi-structured interviews	Thematic analysis
Gajaria et al. (2024) [34]	Canada	Adolescents, caregivers, service providers; *N* = 10	Mental health service; diagnosed with a depressive disorder	14–18	N/a	Focus groups	Thematic analysis
Goo et al. (2019) [35]	Singapore	Adolescents and their parents; *N* = 9	Children’s hospital in central Singapore; met DSM-IV criteria for MDD	14–18	N/a	Semi-structured interviews	Thematic analysis
Kitchen et al. (2022) [36]	United Kingdom	Adolescents and their parents; *N* = 5	Attending public mental health service for children and adolescents in the North East of England for behavioral activation treatment for MDD	12–17	N/a	Semi-structured interviews	Thematic analysis
Liu et al. (2020) [37]	China	Adolescents and their parents; *N* = 12	Attending outpatient hospital departments in Shanghai for MDD	16–18	N/a	Semi-structured interviews	Thematic analysis
McKeague et al. (2022) [38]	Ireland	Parents; *N* = 40 (28 mothers/12 fathers)	CAMHS – adolescents receiving treatment for ADHD or depression	10–16 (13.88)	N/a	Semi-structured interviews	Thematic analysis
Njau et al. (2024) [39]	Tanzania	Caregivers; *N* = 15 (12 mothers; 3 fathers)	Primary HIV care and treatment centers in Dar el Salaam City	12–19 (15.2)	27–61 (45.6)	Semi-structured interviews	Reflexive thematic analysis
Pontes et al. (2022) [40]	Brazil	Parents/primary caregivers; *N* = 26 (23 mothers/3 fathers)	Child Psychosocial Care Centers in Southwrn Brazil(community-based mental health service)	12–18	N/a	Semi-structured interviews	Thematic analysis
Radez et al. (2022) [41]	United Kingdom	Parents; *N* = 20 (18 mothers/2 fathers)	School-based sampling of adolescents in Berkshire who meet diagnostic criteria for anxiety and/or depression	11–17	N/a	Semi-structured interviews	Thematic analysis
Rhodes et al. (2023) [42]	United Kingdom	Adolescents and their parents; *N* = 7 (6 mothers/1 father)	Charities in Scotland and social media; diagnosis of autism and depression	9–18 (15.05)	41–51 (47.67)	Semi-structured interviews	Reflexive thematic analysis
Rose-Clarke et al. (2021) [43]	Nepal	Adolescents, parents/caregivers, teachers, healthcare workers, non-governmental organization representative; *N* = 39	Rural mountainous district in Nepal	11–19	N/a	Focus groups	Framework method
Schlimm et al. (2021) [44]	United Kingdom	Parents; *N* = 16	Multicentre randomized controlled trial in London – parents of adolescents receiving CBT for moderate to severe depression	11–17	N/a	Semi-structured interviews	Thematic analysis
Sibeoni et al. (2022) [45]	France	Adolescents and their parents; *N* = 27 (27 parents − 7 couples/10 mothers/3 fathers)	Attending psychiatric departments for MDD in Paris, Bobigny, Dunkerque, Lille, and Dôle	13–18 (15.6)	N/a	Semi-structured interviews	IPSE (Inductive Process to analyse the Structure of lived Experience) approach
Stafford et al. (2024) [46]	United States	Adolescents, parents and legal guardians, healthcare providers; *N* = 56	Latino/a/x families in the United States recruited via social media, primary and mental healthcare settings	13–17	N/a	Semi-structured interviews	Directed qualitative content analysis
Stapley et al. (2015) [47]	United Kingdom	Parents; *N* = 48	Public mental health service for children and adolescents in North London; referred for moderate to severe depression	11–17	32–64	Semi-structured interviews	Thematic analysis
Stapley et al. (2017) [48]	United Kingdom	Parents; *N* = 33	Public mental health service for children and adolescents in London; referred for moderate to severe depression	11–17 (15.22)	33–64	Semi-structured interviews	Ideal type analysis
Tumbay (2018) [49]	Peru	Fathers; *N* = 5	Adolescents presenting to emergency healthcare services for their first suicide attempt	14–15 (14.8)	36–53 (45.0)	Semi-structured interviews and test of incomplete sentences	Thematic analysis
Viduani et al. (2021) [50]	Brazil	Adolescents, parents, social workers, health workers, educators, and policy makers; *N* = 6 (4 mothers/2 fathers)	State schools in Porto Alegre; lived experience of adolescent depression	14–17 (15.3)	N/a	Interviews and focus groups	Thematic analysis
Wentholt et al. (2024) [51]	Netherlands	Adolescents, two primary caregivers for each adolescent; *N* = 60 (34 mothers; 26 fathers)	Mental healthcare facilities in Leiden	12–18 (15.67)	36–64	Semi-structured interviews	Iterative thematic analysis
Zhang et al. (2020) [52]	China	Primary caregivers; *N* = 12	Inpatient and outpatient hospital departments for MDD	12–18 (15.37)	43.57	Mixed methods - qualitative interviews	Inductive thematic analysis
Zhang and Zhou (2025) [53]	China	Parents; *N* = 7	Parents involved in DuGuo, an online community supporting the families of adolescents with depression in China	14–17	41–55	Mixed methods - Semi-structured interviews	N/a
Zhao et al. (2025) [54]	China	Parent; *N* = 12 (10 mothers/2 fathers)	Child and adolescent psychology outpatient clinic in a tertiary-level hospital in Zhejiang Province, China	14–18	38–50	Semi-structured interviews	Colaizzi 7-step analysis method
Zhou et al. (2025) [55]	China	Parent-adolescent dyads; *N* = 21 (20 mothers/1 father)	Secondary school in North China	14–16	N/a	Mixed methods – semi-structured interviews	Inductive thematic analysis

*N/a* Information not available, *MDD* Major Depressive Disorder, *ADHD* Attention Deficit Hyperactivity Disorder, *CBT* Cognitive Behavioral Therapy

### Quality assessment

Quality ratings for each study are presented in Table 4. Most studies (*n* = 23) met criteria for seven or more items on the 10-item CASP tool, with the remaining two studies meeting criteria for only two [35], and four items [53]; respectively, therefore, most studies were assessed to be of high methodological quality. Common issues across studies included a lack of clarity/detail on researcher reflexivity, with only two studies clearly stating this [45, 46]; four studies did not provide details on the ethical considerations in the study [31, 35, 42, 49]; three studies did not justify their choice of study design [37, 38, 40], and two did not clearly state the research value and implications of their findings [33, 35].

**Table 4:** Quality assessment of included studies

Paper	Aims	Methodology appropriate	Design appropriate	Recruitment strategy	Data collection addresses research issue	Reflexivity	Ethical issues	Data analysis	Statement of findings	Research value
Armitage et al. (2020) [31]	Yes	Yes	Yes	Yes	Yes	No	Can’t tell	Yes	Yes	Yes
Chukwuere et al. (2022) [32]	Yes	Yes	Yes	Yes	Yes	No	Yes	Can’t tell	Yes	Yes
De Silva et al. (2020) [33]	Yes	Yes	Yes	Yes	Yes	No	Yes	Yes	Yes	Can’t tell
Gajaria et al. (2024) [34]	Yes	Yes	Yes	Yes	Yes	Can’t tell	Yes	Can’t tell	Yes	Yes
Goo et al. (2019) [35]	Can’t tell	Yes	Can’t tell	Yes	Can’t tell	No	Can’t tell	No	Yes	Yes
Kitchen et al. (2022) [36]	Yes	Yes	Yes	Yes	Yes	Can’t tell	Yes	Yes	Yes	Yes
Liu et al. (2020) [37]	Yes	Yes	Can’t tell	Yes	Yes	Can’t tell	Yes	Yes	Yes	Yes
McKeague et al. (2022) [38]	Yes	Yes	Yes	Yes	Yes	No	Yes	Yes	Yes	Yes
Njau et al. (2024) [39]	Yes	Yes	Yes	Yes	Yes	No	Yes	Yes	Yes	Yes
Pontes et al. (2022) [40]	Yes	Can’t tell	Can’t tell	Yes	Yes	No	Yes	Yes	Yes	Yes
Radez et al. (2022) [41]	Yes	Yes	Yes	Yes	Yes	Can’t tell	Yes	Yes	Yes	Yes
Rhodes et al. (2023) [42]	Yes	Yes	Yes	Yes	Yes	No	Can’t tell	Yes	Yes	Yes
Rose-Clarke et al. (2021) [43]	Yes	Yes	Yes	Yes	Yes	No	Yes	Yes	Yes	Yes
Schlimm et al. (2021) [44]	Yes	Yes	Yes	Yes	Yes	Can’t tell	Yes	Yes	Yes	Yes
Sibeoni et al. (2022) [45]	Yes	Yes	Yes	Yes	Yes	Yes	Yes	Yes	Yes	Yes
Stafford et al. (2024) [46]	Yes	Yes	Yes	Yes	Yes	Yes	Yes	Yes	Yes	Yes
Stapley et al. (2015) [47]	Yes	Yes	Yes	Yes	Yes	No	Yes	Yes	Yes	Yes
Stapley et al. (2017) [48]	Yes	Yes	Yes	Yes	Yes	Can’t tell	Yes	Yes	Yes	Yes
Tumbay et al. (2018) [49]	Yes	Yes	Yes	Yes	Yes	No	No	Yes	Can’t tell	Yes
Viduani et al. (2021) [50]	Yes	Yes	Yes	Yes	Yes	Can’t tell	Yes	Yes	Yes	Yes
Wentholt et al. (2024) [51]	Yes	Yes	Yes	Yes	Yes	Can’t tell	Yes	Yes	Yes	Yes
Zhang et al. (2020) [52]	Yes	Yes	Yes	Yes	Yes	No	Yes	Can’t tell	Yes	Can’t tell
Zhang and Zhou (2025) [53]	Yes	Yes	Can’t tell	Can’t tell	Can’t tell	No	Yes	Can’t tell	Can’t tell	Can’t tell
Zhao et al. (2025) [54]	Yes	Yes	Yes	Yes	Yes	No	Yes	Can’t tell	Yes	Yes
Zhou et al. (2025) [55]	Yes	Yes	Yes	Yes	Yes	No	Yes	Can’t tell	Yes	Yes

### Meta-synthesis findings

**Fig. 2:**
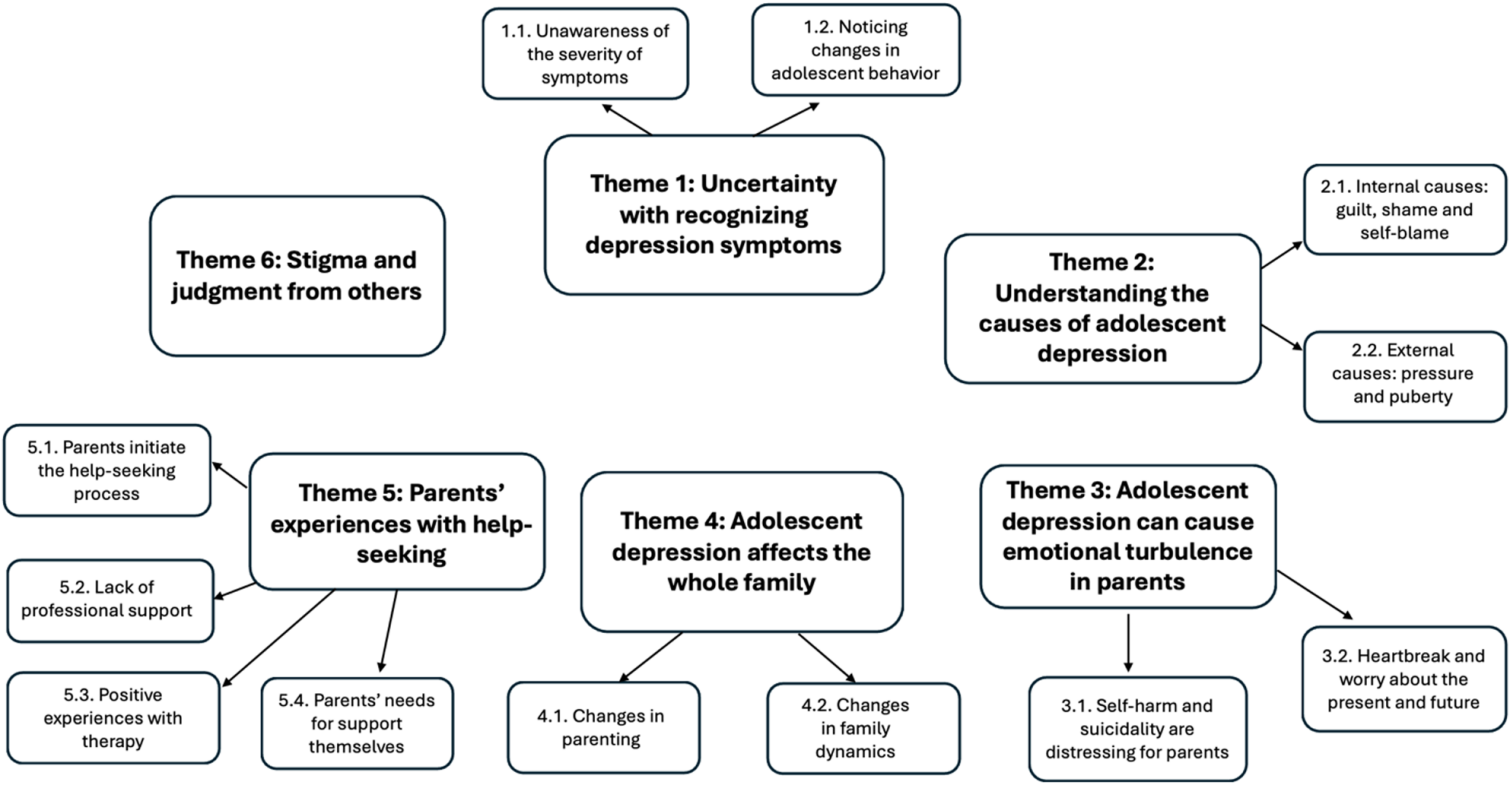
Thematic map

Six overarching themes were generated during analysis, which collectively described parents’ experiences, views, and perceptions of different stages of their child’s depression symptoms, including the initial recognition of symptoms, what was going on for them emotionally, how depression affected the family, and what their thoughts were on the support they received (Fig. 2). Each theme (and subthemes) will be described with the use of quotes from individual papers to highlight parents’ lived experiences.

#### Theme 1: uncertainty with recognizing depression symptoms

The first theme describes the challenges parents face with knowing if or when an adolescent is experiencing symptoms of depression. Two subthemes were generated: unwariness of the severity of symptoms, and noticing that their adolescent’s behavior had subsequently changed.

##### Unawareness of the severity of symptoms

Many parents did not initially recognize that their adolescent was struggling with depression symptoms. For some, the realization only came once the symptoms became more severe and a school or healthcare professional raised concerns. One parent described the stark contrast of not suspecting anything was wrong, and being told suddenly that her daughter had been struggling severely with low mood and self-harm:

*“She went to someone in school and that person at school contacted me and said she’d been to her and that she was unhappy and that she’d been harming herself … I didn’t think there was anything wrong.”* ( [47], p. 622)

For others, their adolescent suddenly disclosed their struggles when their symptoms became more severe.

*“So I came home and I went to see him in his bedroom and he was sitting there crying his eyes out that he feels like a failure*,* that he’s rubbish*,* and that he doesn’t feel that it’s worth living.”* ( [47], p.622)

In three studies, parents reported that they did not think their adolescent’s behavior was unusual considering some low mood was common during adolescence, until they became aware of more severe symptoms [31, 45, 47]. This indicated that while parents acknowledged that some levels of unhappiness could be expected during these years, distinguishing between mood changes associated with adolescence, and depression symptoms, was challenging to balance: *“… I don’t know if that’s a teenage thing or it’s a depression thing”* ( [47], p.623).

Once they did realize their adolescent’s low mood was outside of the usual range of teenage mood changes, parents experienced shock over the discovery; this was particularly overwhelming in cases where the realization came paired with high-risk behaviors such as self-harm [40, 47]. One parent illustrated how upsetting this time was for them.

“*To be honest*,* none of us noticed anything*,* everything happened very fast*,* in a week our life turned upside down. He [adolescent] asked to be hospitalized*,* said he was scared since he had already tried to kill himself. It was the worst thing I have ever heard*” ( [40], p.4) .

##### Noticing changes in adolescent behavior

Parents consistently noticed a change in their adolescent’s behavior, although they were not always able to pinpoint what that change was or what had caused it: “*It’s just not like her. It’s just like having a stranger in the house*” ( [47], p. 621). A father recalled asking himself ‘what was wrong’ after noticing his son had started becoming visibly annoyed more often ( [49], p. 126), illustrating the inner struggle some parents experienced with understanding what may have led to the change in their adolescent’s usual behavior.

UK-based parents described a stark contrast between how their child used to be in the past compared to the present, illustrating how drastic the change was, using past descriptions such as ‘*an absolute joy*’ and ‘*a social bunny’*, and ‘*horrible*,* horrible creature*’ for the present ( [31], p.1623). Others made comparisons about their child ‘before’ and ‘after’, referring to a clear difference in the adolescent’s behavior following the onset of depression [47].

Noticing that their child had withdrawn from the family or social situations was described by parents in six studies: some described how their adolescent would spend increased amounts of time alone in their room [32] and how they would no longer spend time with the family, “*She closed up*,* on herself*,* and we didn’t see her anymore. She spent her time in bed.*” ( [45], p.1422). Some parents may have perceived their adolescent’s withdrawal and lack of energy as ‘*laziness*’, as described by a parent of an adolescent with HIV in Tanzania ( [39], p.16).

Several parents explained how their adolescent’s withdrawal had also isolated them from their friends or made it difficult to maintain friendships and peer interactions, with some highlighting this had not been the case prior to the depression [39, 45, 50]. One parent articulated how this change happened suddenly and led to drastic consequences in the adolescent’s social life:

“*She started to isolate herself socially*,* she was a child who made friends easily*,* everyone adored her. Suddenly she isolated herself until she had no more friends*” ( [40], p. 4).

Therefore, there was a sense of helplessness and confusion accompanying parents’ accounts of observing the behavioral changes in their adolescents, without necessarily being aware of what had been causing them.

#### Theme 2: Understanding the causes of adolescent depression

This theme explores how parents sought explanations for the cause of their adolescent’s depression. While some focused inwardly on their own responsibility as a parent, others looked to external factors which were less in their control. Two sub-themes were generated from the studies.

##### Internal causes: guilt, shame and self-blame

Particularly in two of the UK-based studies [31, 47], a strong sense of shame and guilt was articulated by parents due to the role they believed they played in their child developing depression. Specifically, parents expressed concerns regarding their parenting style and how this may have affected their adolescent’s mental health.

Parents seemed to think that their adolescent had developed depression due to something they did incorrectly with their parenting, or that they failed to be there for them in times of need [32, 37, 38, 47, 52]. These were illustrated through specific recollections of behaviors towards their adolescents which parents now felt guilty about, such as ‘*frustrated shouting*’ ( [47], p.622), inadvertently hindering their adolescent’s independence [37], lacking discipline in their parenting [52], or fighting with their child due to their stubbornness [32]. One parent expressed regret about not listening to their adolescent,*“.you get upset with yourself because you say*,* we weren’t listening to him. He must have been trying to tell us something. We missed this*,* and we missed that*” ( [38], p. 2326), and another blamed their own ‘ignorance and fear’ for the worsening of their adolescent’s symptoms ( [53], p. 8).

Another source of guilt was expressed in a paper including a mother-only sample [31]. These mothers spoke about feeling personally responsible for their adolescent’s happiness, and even that their relationship status or being single mothers ‘*had ruined their child’s life*’ ( [31], p. 1620). The authors of this study pointed out that self-blame for their adolescent depression was expressed in every interview, “*I think as a Mum you automatically feel guilty about everything*,* so I did.*” ( [31], p. 1620), suggesting that mothers felt it was inevitable to blame themselves for their adolescent’s depression.

Some parents who felt a personal responsibility were comforted by receiving psychoeducation on the potential depression being a combination of genetic and environmental factors [38]. This knowledge helped them realize there may have been factors beyond their control that led to their adolescent’s depression.

##### External causes: pressure and puberty

Among parents from five countries, adolescent depression was attributed to external causes, with explicit links made between environmental factors such as bullying, peer interactions, or difficulties in school, and their adolescent’s depression [33, 40, 42, 51]. Some of these external causes were more or less under a parent’s control. For example, one parent described switching their child to a different school to try and stop the bullying:

“*She told me that they bully her*,* her classmates*,* at school and that caused her problems like falling into depression more and more. So*,* I changed her from that school; I do not like it at all*” ( [32], p.6).

Although puberty is an internal cause to the adolescent, to parents it is external, and a factor which parents may feel is entirely out of their control. Across six studies the subject of depression in relation to typically occurring changes in puberty was articulated but from several perspectives. Some parents thought their adolescent’s symptoms were linked to a ‘chemical imbalance’ or hormonal changes ( [31], p. 1621) due to the adolescent age, “*…first of all I think her age and hormonal fluctuations and stuff*” ( [51], p. 487). Some wondered whether the depression symptoms were a more extreme version of the typical mood swings that adolescence brings [47], while others were questioning [31] or reassuring themselves that this was a phase that was “*normal*” and “*will pass*” ( [45], p.1422; [49]); similarly a parent in Nepal expressed confidence that depression in adolescence is typical for this stage of life and not something that should be feared [43].

#### Theme 3: adolescent depression can cause emotional turbulence in parents

Parents described a range of experienced emotions during the different stages of their adolescent’s depression. The most prominent one was a fear of their adolescent self-harming, although worry about the short- and long-term consequences of depression on their adolescent was also present. This is explored in two subthemes.

##### Self-harm and suicidality are distressing for parents

The most commonly expressed causes for worry among parents were self-harm and suicidal ideation or feelings. This was highlighted in six studies where parents described their fear and distress over the possibility or risk of their child self-harming or having suicidal thoughts. Some spoke of their worry affecting their daily life through constantly ‘*being on the knife edge*’ by checking their phone at work, being unable to sleep, waking up thinking about their child’s safety ( [31], p. 1621). Researchers in two studies reported that parents were visibly distressed, tearful, and struggled to talk about this fear in the interviews [31, 47]. Similarly, Zhang et al. [52] described that most caregivers in their study had a fear of their adolescent having suicidal ideations. As such, it was clear that parental fear over self-harm had a profound negative impact on their own wellbeing.

While it was not always clear whether parents were referring to a fear of self-harm in adolescents who had not attempted it before or a those who had, in four studies parents were directly referring to a fear of relapse [40, 49, 52]. A parent in France described the added seriousness of their adolescent’s struggles following self-harm:

“*It’s at that moment when you realize that she’s in danger that she could have died. There*,* you have to face the facts*” ( [45], p.1422).

A UK-based mother clearly conveyed her distress over the thought of her child self-harming:

“*The thought of my beautiful child marking her body in that way just distresses me to beyond anything really. I can’t even put into words how distressed I was over it*” ( [47], p.622).

Similarly, a South African parent described the disbelief when she found out that her daughter had attempted suicide, articulating their concern that this should not have happened whilst she was in treatment:

“*I only realized when she attempted the first suicide*,* this time when she went to consult a psychologist*,* she told the psychologist how she attempted to kill herself and I was so shocked what if she killed herself*,* is the treatment not working?*” ( [32], p.4).

These quotes illustrate the gravity of the distress parents experienced over their adolescent self-harming. Some parents also faced the prospect of the worst-case scenario of their adolescent’s self-harm or suicide attempt, and clearly took the matter with utmost seriousness, while simultaneously pointing towards the negative impact on their own wellbeing.

##### Heartbreak and worry about the present and future

In addition to the fear of self-harm, there was a range of other emotions parents articulated as a result of their adolescents struggling with depression. Some were related to the unfolding experience of their child’s symptoms and describing it as ‘*heartbreaking*’ ( [31], p. 1623). In this study, the authors observed that the majority of their sample of mothers either expressed or showed signs of distress. Similarly, a father vividly described how upsetting it was to see his daughter go through hospitalization due to her depression:

“*Then we took her to the hospital*,* we interned her for the first time and that internment to me broke my soul because to take away my daughter in those circumstances*” ( [49], p. 128)

An articulation of worry over their adolescent’s future was also present. A UK-based mother explained she was worried not only about her daughter’s decreased motivation in school, but also how this could extend into adulthood and affect her future career [31]. Parents in two studies from China had similar long-term concerns their adolescents:

“*I worry about her (sick daughter) illness if not cured on the end. I worry about her life*,* study*,* and later future into the society*.” ( [52], p. 1324)

“*Now I am just afraid that this disease of his will keep recurring and stay with him for the rest of his life.*” ( [54], p. 3).

In these studies, parents referred to depression as a medical illness, highlighting intense worry over a permanent or incurable problem which will persist into the adolescent’s future.

#### Theme 4: adolescent depression affects the whole family

This theme captures the effects of adolescent depression on the whole family through two subthemes: changes in parenting to avoid making symptoms worse, and changes in everyday family dynamics to adapt to the adolescent.

##### Changes in parenting

Parents reported the adaptations they made to their parenting style in response to their child’s depression symptoms, although this was done in contrasting ways. As described in Theme 2, some parents felt their parenting was related to their adolescent’s onset of depression, so these changes could have been an attempt to address this. Some described adjusting how strict they had previously been, and found it important to be kinder to their child, while still maintaining some boundaries:

“*I try being reasonably firm with boundaries*,* I try being a bit more lenient*,* a bit kinder. So I’m constantly sort of playing around trying to do the right thing*,* whatever that is*” ( [47], p. 623).

Others felt their parenting was harsh or neglectful of their adolescent’s emotional struggles, but were not sure of alternative ways to approach their communication:

“I do not know how to help her with her emotions. Because I’m not great at communicating, I usually end up making things worse when I try” [55].

Therefore, due to the fear of worsening their adolescent’s wellbeing, some parents may struggle to implement changes to their parenting and communication despite feeling stuck and helpless with the current dynamic they have with their child.

Similarly, there was also a sense of struggling to balance the upkeep of rules and being gentler, which was a change to their parenting in response to the adolescent’s mental health struggles. In several papers, parents used the expressions ‘*walking on eggshells*’ or in one, ‘*tiptoeing around*’ their child to describe this challenge and illustrate how uncertain they felt about their child’s potential responses and reactions to their parenting behavior [35, 47]. In some cases, parents minimized giving their child instructions:

“*We have changed some interactions or different way we talk to her… we don’t tell her many things the way we used to do… For example*,* about*,* mostly about the reading. Go for study and do that*,* we don’t do that. She doesn’t like the straight approach. Even if you ask her result*,* she doesn’t like.”* ( [35], p. 22).

In contrast, some imposed stricter rules due to the fear of their adolescent self-harming if they went out unaccompanied [54]. Across the different approaches parents took to changing their parenting, they conveyed the vast amounts of effort they put into ensuring they did not trigger a worsening of symptoms or overwhelming their adolescent.

##### Changes in family dynamics

Parents reported that their adolescent’s depression affected the dynamics of the whole family. This was particularly noticeable due to a reduction in the activities and amount of time their child was spending with the family compared to the past [31]. Some parents responded to this by adapting their family plans and routines to help their child with depression:

“*We have to sort of plan things differently so that he is not alone … he’s so sad sometimes I don’t want him to feel like we’re going off and abandoning him*” ( [47], p. 625).

Some parents expressed how ‘*draining*’, and ‘*mentally and physically exhausting*’ it had been to provide additional parenting to their adolescent with depression. They indicated that this had been affecting their capacity to show up as a parent, in some cases to their other children, and in their own day-to-day activities such as work, implying there was a degree of parental burnout and low energy levels [47, 53, 54]. This was echoed in another study where authors attributed the reduction of usual family life to parents’ ‘*depleted emotional stamina*’ in addition to the lack of energy and time while caring for their adolescent ( [52], p. 1324).

Conversely, other parents expressed that supporting their adolescent led to positive changes within the family. These included an improvement in their parenting skills and resilience [54], family closeness [40], or brought on a ‘*temporary calm’* in the family ( [37], p. 6). Some described positive changes to their marriage [40, 54], putting issues aside to focus on helping their adolescent:

“*I always had some disagreements with my husband. However*,* this time my baby is sick. It is also a good time for me to talk with my husband.*” ( [52], 2020, p. 1324).

A father who was usually less present at home described becoming more involved in family life and becoming more flexible in his day-to-day life once his daughter had started experiencing depression symptoms:

“*That is why I do not leave her*,* that is why I support her. My schedule at work has changed*,* I do not have a fixed schedule*,* my family is more involved*,* I am with my children.”* ( [49], p.129).

Therefore, while changes to family dynamics were evident, parents could either experience them as negative – feeling unable to show up as a parent as they usually do and making careful adjustments at home to minimise distress to their adolescent, or positive, whereby supporting an adolescent with depression made families grow closer in a joint effort to provide additional care to their family member.

#### Theme 5: parents’ experiences with help-seeking

This theme captures the range of experiences parents had with help-seeking for their adolescents. While parents were often the ones initiating the process, satisfaction with the support received varied from feeling misunderstood by healthcare professionals, to positive views on therapy for the adolescent. Finally, parents also identified their own needs for receiving support themselves.

##### Parents initiate the help-seeking process

Parents in seven studies expressed taking the initiative to seek help for their adolescent. For example, UK-based parents described seeking professional and online resources to increase their understanding of their adolescent’s symptoms and struggles so that they can support them better [47]. Some parents sought the help of a mental health professional who acted as a mediator between them and their child to facilitate better communication between them [45].

Similarly, some parents described suggesting external support to their child in hopes of them feeling able to open up more to someone outside of the family. This included parents balancing between being supportive and open to talking to their child while also not being intrusive, giving them space and autonomy in their therapy process. For example, several parents said they would not actively ask their child what they were discussing in therapy [44], but would welcome their child sharing information from their sessions:

*“[I tried] to give him a lot of space for himself*,* you know*,* support him… I know that he feel very awkward talking to me with his problem*,* so I say it’s ok that you don’t tell me but you have to speak to someone*,* whoever you feel you can trust…*” ( [35], p. 21)

Beyond approaching mental health professionals, some turned to the adolescent’s school when not knowing how to help, explaining and that seeking help with school professionals was a collaborative effort [41, 54]:

*“…so me and my husband went into school and said ‘look*,* we need help*,* what do we do’*,* obviously we didn’t*,* I wouldn’t know how to deal with something like that and they basically helped us and we helped them*,* if you know what I mean*,* so we worked together*” ( [41], p. 902).

While some parents sought formal support by approaching professionals on the adolescent’s behalf, some used informal avenues such as encouraging the adolescent to make lifestyle changes which could lead to a positive impact to their mood. Specifically, Latino parents in the US described suggesting physical activity such as sport, or initiating walks with their adolescent as a way to help them feel better:

“*…I take her with me on a walk… we don’t take the bus*,* we walk so she relaxes. And I tell her*,* ‘let’s go!’ And she*,* but when I start to see that she calms down*” ( [33], p. 8).

##### Lack of professional support

Parents felt their child was misunderstood by mental health professionals, with some expressing a concern that their child was not being treated well in these settings:

“*I do not just understand how they operated because I think they were against her like they were not treating her fine; they were disrespectful*,* because I used to tell them what was happening but they would not listen to me…they were not listening to what I was telling them about my child‘s situation.*” ( [32] p. 4).

This was accompanied by a sense of frustration due to the parent feeling left out of the process, despite feeling that they had important insight about their adolescent which could have been important incorporate into the treatment.

There was a sense of parents’ expectations for their child’s therapy not being met, or that therapy was not helpful:

“*I did take her to a therapist and I*,* we kind of jumped around cause I just didn’t really feel like she was getting what she needed out of it. Like I’m like tools*,* I want tools*,* like I don’t want my daughter to go sit down and just talk about her feelings…”* ( [33], p. 8).

UK-based parents in two studies seemed to be disheartened by healthcare professionals, including their general practitioner (GP) not listening to their concerns [47], or making an effort to understand the day-to-day implications of adolescent depression, “*Yes they understand the textbook version but you don’t actually understand living it which is totally different*” ( [31], p. 1623).

##### Positive experiences with therapy

In contrast to the frustration and negative experiences above, other parents felt that they were well supported, particularly by healthcare professionals. They reported noticing progress as a result of therapy [32] and that therapy had ‘*brought their child back*’, alongside a strong sense of relief now that their child’s ‘old self’ was re-emerging, *“She’ll join in with the conversations….and…waffle the whole time…she’s not sitting up there sulking anymore.”* ( [44], p. 1024).

While UK-based parents felt barriers in accessing support through their GP, in the Latin American community in the US, the pediatrician was described as a facilitator in referring the child to further support [33].

Parents who had positive experiences with support continued to use ‘then’ and ‘now’ comparisons as highlighted in Theme 1, alluding to the perception of change that depression had caused but that therapy was improving. Some were pleased with the tools and knowledge their child had gained in cognitive-behavior therapy:

*‘He can make decisions now…he used to have them made for him…through his therapy he’s learnt the ways of thinking about things’. (* [44], *p. 1024).*

Parents thought that the success of their child’s therapy was largely dependent on the individual therapist and their therapeutic relationship with the child. They acknowledged that the quality of the adolescent-therapist relationship may be based on the open communication which the adolescent may not feel comfortable having with a family member.

“*It’s always difficult when it’s family…you don’t want to upset them…whereas when you’re talking to a therapist…you can just say exactly what’s on your mind.” (* [44], *p. 1025).*

##### Parents’ needs for support themselves

Parents expressed the need to receive support for their own wellbeing, including the lack of energy and burnout described in Theme 2, their parenting style, and ways to support their child at home [33, 44, 47]. There was a desire for more psychoeducation on depression symptoms both from online and in-person interactions with healthcare professionals [33, 54] and curiosity about its causes and symptoms [37]. Parents also conveyed an interest in ways and strategies to support their child’s recovery process outside of therapy [33, 39, 44, 47), as well as ways to parent and enforce rules while their child was recovering [47].

Those who conveyed the feeling of being lost and alone explained it would be helpful to know they are not the only ones struggling in this way [47] and that it would be reassuring to hear from other parents who have been through a similar experience:

*‘‘I would like to chat with other moms that have had children with depression and who have already come out or are much better than when they started with this situation”* ( [46], p. 8*)*

Additionally, there was a desire for parent-directed resources to be provided by healthcare professionals, with some parents stating that it was not enough for their child to be receiving support on their own [31, 47]. One parent suggested a specific example of how this may look:

*“That there would be classes for parents. Classes where they can teach them about the stages of life in children*,* how sometimes children can be sad*,* mad*,* and all the things that change with them.” (* [33], *p.9).*

#### Theme 6: stigma and judgment from others

Stigma and judgment from others were directly described by parents in most studies and across cultures.

Parents worried that their adolescent receiving a diagnosis could lead to stigma, indicating that they were not comfortable with the ‘label’ of a mental health problem being linked to their child in the long-term: “*I wouldn’t want her to be diagnosed with something long-term either*,* bi-polar or sort of.*” ( [31], p. 1621). In the Latin American community in the US, parents had serious concerns that healthcare professionals’ judgment over their parenting would lead to them losing rights over their child, which was also a barrier to seeking treatment [33].

Some were concerned about others, including teachers and other parents, judging them for their parenting and blaming their child’s depression symptoms on this. For example, two parents in Ireland perceived that other adults attributed the adolescent’s depression to their parents ‘spoiling’ and ‘babying’ them, and that they would make intolerant comments such as “*Jesus I don’t know how you’re living with someone like that”*, referring to the adolescent with depression ( [38], p. 2325). These opinions contributed to parents feeling responsible for their child’s struggles [38]. Parents also said that judgments from other adults would sometimes target the adolescents themselves, for example, by attributing the depression to adolescents being lazy or spoilt [33, 38].

Parents in Peru [49], China [54], and those from the Latin American community in the US, spoke of having to hide their adolescent’s depression, “*And also*,* Latinos hide if you have a child with any given condition; you always try to hide it.*” [33, p. 7). These accounts highlighted the range of cultural beliefs about adolescent mental health across countries, and how they prompt parents to behave differently to avoid judgment. Latino parents explicitly spoke of mental health stigma in their community. They seemed to have been aware of how people from their background did not take adolescent depression seriously, often attributing it to behavior such as ‘*laziness*’ or the child ‘*acting crazy to not help at home*’ ( [33], p. 6).There was a belief that ‘*at least half*’ of people in their community held the views that taking one’s child to a psychologist or psychiatrist means the child is ‘*crazy*’ ( [33], p. 7). Parents in this study also thought these views were embedded in the Latino culture and passed down from older generations. It was unclear whether these parents were distancing themselves from the stigma in their community since they identified it as unhelpful, or if elements of self-stigma were simultaneously present amongst them nonetheless.

In another study, parents were self-reflective about their own preconceptions about their child’s symptoms and how these changed; for example, a parent in China described how a therapist helped them accept their child’s depression instead of seeing it as ‘*pathological*’ or abnormal ( [37], p. 5). This could indicate that even when mental health stigma is present in a community, once a parent faces a personal experience such as supporting their adolescent with depression, the stigma becomes present or reduced with the right information provided.

## Discussion

### Summary of findings

This systematic review of 25 qualitative studies including 523 participants synthesized parents’ experiences of adolescent depression across 13 countries. Six themes were generated. These described parents’ initial struggles with identifying and coming to terms with the changes in their adolescent brought on by the depression, and the emotional toll this took on the parents, particularly distress relating to adolescent self-harm. The whole family became affected, including changes in family dynamics to adapt to the adolescent with depression, and parents described being more lenient with their parenting to avoid making symptoms worse. Experiences with professional support varied, with some noticing positive changes in their adolescent following therapy, and some feeling a lack of understanding from healthcare professionals. Finally, parents spoke of their fear of or experiences with stigma from healthcare professionals and other parents which for some acted as a barrier to accessing support.

Problem recognition is a key part of the help-seeking process in Rickwood and Thomas’ [17] help-seeking model, and across the papers included in our review, parents’ accounts highlighted difficulties with recognizing adolescent depression symptoms. Specifically, there were mixed accounts of the initial stages of the child’s depression, with some parents not recognizing the symptoms until they got more severe, and others noticing their adolescent was not being their usual self. This could explain why access to treatment for adolescents is delayed when parents do not recognize the symptoms [12, 13]. This lived experience is also in line with findings on limited mental health literacy among parents [19], showing from first-hand accounts that increased psychoeducation on early adolescent symptoms is needed for parents. Distinguishing between the usual changes in adolescent behavior and mood, and depression symptoms posed a further challenge to the recognition of symptoms which can be expected due to the overlap between the age of depression onset and physiological changes as puberty advances [56].

Across the synthesized studies, once parents recognized the problem, they sought explanations and causes of their child’s depression. While some felt personal responsibility due to their parenting style, accompanied by feelings of guilt and regret, others placed more importance on social or biological factors beyond their control, such as bullying and hormonal changes. Parents’ accounts of self-blame and attributing their child’s depression to their own shortcomings as a parent are in line with previous quantitative findings showing that 60% of the adolescent parent sample indicated partial or full responsibility for their child’s mental health problem [57]. Consistently with the findings in this review, the causal beliefs adolescents hold about their own depression vary between psychosocial factors such as bullying and peer relationships, to biological factors such as genetic and hormonal changes [20, 58]. While parents in the present review tended to report self-blame due to their parenting or own mental health, adolescents expressing self-blame are those who attribute their depression to biological factors. Despite similar factors coming up both among adolescents and parents, further research is needed to understand how these overlap, and how and whether parents and their children communicate about them.

Parents’ own wellbeing was affected by their adolescents’ struggles. There was a sense of sadness and shock upon realizing their adolescent had been experiencing depression symptoms, and noticing how the adolescent was acting in the present compared to the past. The prospect of their child self-harming or relapsing if they had already done so in the past had an additional negative emotional impact on the parents, which has also been documented in a meta-synthesis exploring parental experiences with child and adolescent self-harm specifically [59]. It is worth noting that there was no overarching cause of parental distress about other features of adolescent depression, highlighting the negative emotional impact that self-harm, in particular, can have on parents. Beyond affecting the parents themselves, they recounted how the adolescent depression affected the whole family.

Together, themes three and four can be interpreted in the lens of the family systems theory, which posits that families can be understood as a cohesive unit whose members have mutual, and sometimes unintended, influence on one another [60]. The theory particularly focuses on the complex dynamics leading to emotional and behavioral consequences within families. While adolescent depression may affect parents negatively through the emotional distress and additional efforts to adapt their parenting, parents in turn may experience burnout, leading to decreased parental involvement and warmth; both of these factors are associated with increased adolescent depression symptoms and decreased parental help-seeking behavior [61]. Although most research so far has focused on the effects of negative family functioning and parenting styles on adolescent depression [62], some longitudinal research drawing on the family systems theory shows that a reciprocal relationship between them may be at play [63], which this meta-synthesis can corroborate from parents’ lived experiences perspectives.

From the synthesized studies, it was clear that parents initiated the help-seeking process and were driven to help their adolescent, which is line with the knowledge that parents play a key role in accessing support or providing it themselves to their children [64]. These findings deepen this knowledge with lived experience perspectives, demonstrating that parents proactively seek various ways to support their adolescent, including online and in person through healthcare and educational professionals. However, experiences with professional support varied. Some felt misunderstood and not heard by their GPs, which reflects the views or some adolescents who felt ‘let down’ by CAMHS [21]. Other parents noticed a positive change and their adolescent returning to their usual self as a result of therapy, which aligns with quantitative findings on relatively high parental satisfaction with CAMHS for mental health problems in general [64].

Finally, parents across different countries spoke of their fear or direct experiences of stigma towards adolescent mental health problems. These ranged from the concern that a diagnosis would lead to long-term stigma from healthcare professionals, to being judged and blamed by fellow parents for their adolescent’s depression. These findings may be explained in the context of stigma by association, referring to a sense of shame, blame, and judgment towards the relatives of those struggling with mental health problems [65]. In addition to perceived stigma from others, quantitative evidence shows that 20% of parents may experience internalized stigma [57], also known as self-stigma, whereby they attribute negative stereotypes to themselves, and experience fear and shame in anticipation of being discriminated for their own or their adolescent’s mental health problems. In parents, this is correlated with their intention to conceal their child’s mental health problems form others, which may become easily observed by the adolescent and thus contributing to their own internalized stigma [57]. Adolescents’ lived experiences of depression also describe self-stigma, such as the belief that depression “is not an acceptable form of mental health problem” [21]. This type of stigma in adolescents could be a contributing factor to the concealment of symptoms, which in turn make it more difficult for parents to identify symptoms and initiate help-seeking, even among those with higher mental health literacy. Therefore, the influence of internalized and perceived stigma both among parents and adolescents, as well as how these interact, is likely to be complex. The present findings emphasize the already established need for interventions targeting stigma reduction for adolescent mental health, which is a known barrier to accessing treatment [16].

### Strengths and limitations

#### Strengths and limitations of the current review

To the knowledge of the authors, this is the first systematic review to synthesize qualitative evidence on parents’ experiences with adolescent depression. The strengths of the review include the use of an established methodology and systematic search approach reported in line with ENTREQ [22] (Additional File 2) and PRISMA [23] guidelines (Additional File 1), and pre-registration of the review protocol to increase transparency. The authors engaged with reflexivity throughout the meta-synthesis process to ensure they were aware of how their own experiences, values and beliefs may influence the findings. A parent advisor was consulted to ensure that the findings are appropriate and reflective of the experiences of parents of adolescents. The literature search yielded studies from Europe, North and South America, Asia, and Africa, thus capturing the experiences of parents beyond the Western setting; however, despite the range of countries included, cultural context and nuance in individual settings could become lost during the synthesis process.

Studies were included if depression was the primary focus, or where depression was studied in relation to co-morbid mental, neurodevelopmental and physical health conditions, with the aim of reflecting the voices of parents who may be supporting a child with multiple health problems. Whilst this promotes validity in the real world where most adolescents with depression also have other comorbidities [66], what we cannot do is separate out from the narratives of parents which components were specific to adolescents’ struggles with depression. Our findings indicated that parents supporting an adolescent with a physical health issue (HIV; [39]) in addition to depression may experience unique challenges, and further insight is required into these experiences to understand the specific needs of these parents and families.

Further limitations of this review include the exclusion of grey literature, meaning that smaller-scale qualitative studies such as dissertations are not represented. This limits the comprehensiveness of this review and could mean that there is the potential for publication bias. Including only studies in English, Italian and Spanish, and double screening only 30% of the papers for pragmatic reasons were further limitations, although inter-rater reliability was high. These limitations mean that there is a chance that some studies which should have been included (e.g. in other languages) may have been missed.

Two studies [33, 42] included parents of children who were younger than 10 years old. These studies were still included in the review as the mean ages of the children fell into the age range specified in the inclusion criteria and had the potential to provide relevant insight into the phenomenon under study. While an option could have been to only analyze quotes provided by parents of children within the specified age range, this was not reported, and it was not deemed feasible to request the raw data from authors. Subsequently, the study findings should be interpreted with this in mind. Finally, these findings should be interpreted in light of the reflexivity statement.

#### Strengths and limitations of the included studies

The included studies were generally high quality; however, most included studies did not provide information on reflexivity which limits the understanding of how authors’ own attitudes, assumptions and backgrounds may have influenced the generation and interpretation of themes in the studies. Most studies included parents of adolescents through mental health services which provides consistency regarding the insight into the experiences of those whose children’s symptoms require professional support. However, this also means that our findings may not capture the experiences of parents who do not seek support or who are in the early stages of seeking support or whose children are experiencing early symptoms of depression.

### Clinical implications

Table 5 shows key clinical implications across the identified meta-themes and subthemes. Increased psychoeducation is needed on the causes of adolescent depression and how to support their child in addition to professional help. Parent-directed online programs [67] and involvement in therapist-delivered intervention for depression are available [68], as well as CBT-based guidance for parents on supporting an adolescent with depression [69]. Nonetheless, developing approaches to make these more scalable may be needed in order to reach parents on a wider scale who have expressed the need for support with supporting their adolescent. Despite parents showing initiative to find support avenues, meta-syntheses of adolescents’ own experience show that adolescents feel a lack of parental support and involvement [20, 21], suggesting that there may be a communication barrier between them, or that parental approaches to supporting their adolescents may not be directly addressing their needs. Therefore, psychoeducation on parent-adolescent communication or healthcare professionals supporting families with improvements in this aspect could be beneficial.

**Table 5:** Clinical implications across meta-themes

Meta-Synthesis Theme	Outcomes Identified / Sub-Themes	Clinical Implications / Recommendations
Theme 1: How do you know when your adolescent has depression?	· Parents struggle to recognize symptoms of adolescent depression · Parents notice changes in adolescent behavior but struggle to contextualize / understand these changes and what they mean	· Increasing availability and access to parental psychoeducation programs / information about adolescent depression is crucial – particularly focusing on early symptom recognition; differentiating between teenage mood changes and depression symptoms
Theme 2: Understanding the causes of adolescent depression	• Some parents attribute their adolescent’s depression to their own parenting shortcomings and feel blame, shame and guilt for this • Others attribute the depression symptoms to factors beyond their control – such as peer interactions and hormonal changes during puberty	• Provide support for parents who experience negative feelings around self-blame • Offer psychoeducation on known factors contributing to adolescent depression and their multifaceted nature
Theme 3: Adolescent depression can cause emotional turbulence in parents	• Finding out about their adolescent self-harming or showing suicidal ideation can be very distressing to parents • Parents experience sadness about their adolescent’s struggles with depression, and worry about their future and long-term prospects	• Parents’ own wellbeing can become negatively affected when supporting an adolescent with depression. • Mental health professionals may want to explore this issue and support options as parental mental health issues may worsen adolescent mental health symptoms (bi-directional relationship)
Theme 4: Adolescent depression affects the whole family	• Parents describe having to adapt their parenting (e.g. more leniency, or more strictness in some cases) in response to their adolescent’s depression • The wider family may have to adapt to the adolescent, including changes to usual family activities and communication	• Important to support primary caregiver(s), especially those who may have further caring responsibilities. • Provide a whole family support approach to help family members navigate and understand what the adolescent with depression may be going through, and also how to look after themselves
Theme 5: Parents’ experiences with help-seeking	• Parents often initiate the help-seeking process • Parents may experience mixed views (negative / lack of support vs. positive) with therapy and seeking support • Parents supporting a depressed adolescent need support for themselves	• Parents can be identified as key players in help-seeking for adolescent depression • Identifying “therapeutic fit” is key when help-seeking, which may mean trialing several therapeutic approaches or providers to identify the right “fit” for each adolescent • Parental self-care and additional support are important. Parents should seek out their own mental health support services or support groups.
Theme 6: Stigma and judgment from others	• Parents worry that stigma around mental health diagnoses can negatively affect their adolescent’s future – whether it is from healthcare professionals, teachers, or their community • Some parents emphasized particular experiences of stigma in different cultural contexts	• Stigma may pose a barrier to parents seeking timely treatment from healthcare services or school professionals • It is important for mental health professionals to be aware that stigma may vary across communities and cultural contexts • Some parents may have their own prejudice and stigma towards adolescent mental health problems, which could be addressed with mental health professionals

Themes 3 and 4 suggest a whole family approach to support may be beneficial due to the effects of adolescent depression spreading to the wider family and beyond the primary caregiver. This enhanced support provision could involve improved communication and information sharing between different professionals supporting family members separately, offering improved jointed care. Some mental health charities offer information tailored to different family members, acknowledging the family-wide impact on adolescent mental health. Mental health services may benefit from adopting a similar approach to providing family support.

Because of parental accounts of feeling misunderstood or under-supported by GPs and therapists in Theme 5, it is important for healthcare professionals to be informed on the importance of involving parents, their role in supporting an adolescent with depression, and what they struggle with while doing so.

These findings highlight from a lived experience perspective that the delay in parental help-seeking for their adolescent may stem from the symptom recognition stage, and particularly the difficulties with distinguishing between typical changes occurring in adolescence, and symptoms of depression.

### Research implications

Future qualitative studies should follow appropriate reporting guidelines, such as those outlined in Braun and Clarke’s reflexive thematic analysis [70, 71], and specifically, should provide context on the researchers’ reflexivity and positionality in the interpretation of qualitative data to increase the understanding of how their background and theoretical assumptions shaped the generation of themes.

All studies which included parental gender consisted predominantly of mothers, except for one study which had a father-only sample [49]. Fathers are consistently underrepresented in adolescent mental health research [72]. Together with the findings that fathers are often unaware of their role in managing their child’s depression symptoms [73], it is crucial that more studies explore fathers’ experiences with adolescent depression so that they can inform fathers’ support needs and improve psychoeducation for this group. Beyond fathers, including other caregivers who might take on a parental role, such as adoptive parents, grandparents, or foster carers, would also provide more insight into the experience of those who are not biological parents and as such may experience unique challenges in supporting an adolescent with depression.

The studies in our meta-synthesis predominantly included samples of parents who have already supported their child through accessing mental health services; therefore, future studies should seek to understand the views of parents who may be experiencing more barriers to accessing support so we can understand how parents experience these.

## Conclusion

To conclude, this qualitative meta-synthesis is the first to combine multiple accounts of parents’ experiences of their adolescent child’s depression. These themes capture the multifaceted nature of parents’ own journeys of supporting an adolescent with depression. Parents experience uncertainties from the early stages of recognizing symptoms and understanding causes, to negative effects on their own wellbeing due to fear and worry for their adolescent’s mental health. These findings emphasize the importance of understanding parents’ needs and lived experiences, which could inform a range of potential avenues for supporting adolescents through their parents, as well as parents’ own wellbeing.

## Supplementary Information

Supplementary Material 1.

Supplementary Material 2.

Supplementary Material 3.

Supplementary Material 4.

## Data Availability

Secondary data from individual papers were analysed. These can be accessed through the reference provided for the included papers.

## Abbreviations

ADHD: Attention Deficit Hyperactivity Disorder
CAMHS: Child and Adolescent Mental Health Services
CASP: Critical Appraisal Skills Programme
CBT: Cognitive Behavioral Therapy
ENTREQ: Enhancing transparency in reporting the synthesis of qualitative research
GP: General Practitioner
MDD: Major Depressive Disorder
PRISMA: Preferred Reporting Items for Systematic reviews and Meta-Analyses
