# Supplementary material for: Parents’ experiences of their adolescent child’s depression: a qualitative systematic review and meta-synthesis
Source: BMC Psychol. 2026 Jan 9;14:188. doi: 10.1186/s40359-025-03923-2 (PMC12882447; doi:10.1186/s40359-025-03923-2)
Supplement: Supplementary file 2 — Supplementary Material 2. [file 40359_2025_3923_MOESM2_ESM.docx]

## Enhancing transparency in reporting the synthesis of qualitative research: ENTREQ Checklist (Tong, *et al.,* 2012)

| Item No. | Guide and Description | Report Location |
| --- | --- | --- |
| 1. Aim | State the research question the synthesis addresses | Title, abstract – p1 |
| 2. Synthesis methodology | Identify the synthesis methodology or theoretical framework which underpins the synthesis, and describe the rationale for choice of methodology (e.g. meta-ethnography, thematic synthesis, critical interpretive synthesis, grounded theory synthesis, realist synthesis, meta-aggregation, meta-study, framework synthesis) | Introduction – p5 |
| 3. Approach to searching | Indicate whether the search was pre-planned (comprehensive search strategies to seek all available studies) or iterative (to seek all available concepts until they theoretical saturation is achieved) | Abstract (p1), Search strategy (p5) |
| 4. Inclusion criteria | Specify the inclusion/exclusion criteria (e.g. in terms of population, language, year limits, type of publication, study type) | Inclusion criteria, Table 1 – p6 |
| 5. Data sources | Describe the information sources used (e.g. electronic databases (MEDLINE, EMBASE, CINAHL, psycINFO), grey literature databases (digital thesis, policy reports), relevant organisational websites, experts, information specialists, generic web searches (Google Scholar) hand searching, reference lists) and when the searches conducted; provide the rationale for using the data sources | Search strategy – p5 |
| 6. Electronic Search strategy | Describe the literature search (e.g. provide electronic search strategies with population terms, clinical or health topic terms, experiential or social phenomena related terms, filters for qualitative research, and search limits) | Search strategy, table 2 – p7, Additional File 3 |
| 7. Study screening methods | Describe the process of study screening and sifting (e.g. title, abstract and full text review, number of independent reviewers who screened studies) | Study selection (page 8), (Figure 1 PRISMA - flow diagram (p13) |
| 8. Study characteristics | Present the characteristics of the included studies (e.g. year of publication, country, population, number of participants, data collection, methodology, analysis, research questions) | Study characteristics, Table 3 (p15) |
| 9. Study selection results | Identify the number of studies screened and provide reasons for study exclusion (e.g. for comprehensive searching, provide numbers of studies screened and reasons for exclusion indicated in a figure/flowchart; for iterative searching describe reasons for study exclusion and inclusion based on modifications to the research question and/or contribution to theory development) | Figure 1 - PRISMA flow diagram (p13) |
| 10. Rationale for appraisal | Describe the rationale and approach used to appraise the included studies or selected findings (e.g. assessment of conduct (validity and robustness), assessment of reporting (transparency), assessment of content and utility of the findings) | Quality assessment – p9 |
| 11. Appraisal items | State the tools, frameworks and criteria used to appraise the studies or selected findings (e.g. Existing tools: CASP, QARI, COREQ, Mays and Pope [25]; reviewer developed tools; describe the domains assessed: research team, study design, data analysis and interpretations, reporting) | Quality assessment – p9  CASP |
| 12. Appraisal process | Indicate whether the appraisal was conducted independently by more than one reviewer and if consensus was required | Quality assessment – p9 |
| 13. Appraisal results | Present results of the quality assessment and indicate which articles, if any, were weighted/excluded based on the assessment and give the rationale | Quality assessment – p9,  Table 4 (p28) |
| 14. Data extraction | Indicate which sections of the primary studies were analysed and how were the data extracted from the primary studies? (e.g. all text under the headings “results /conclusions” were extracted electronically and entered into a computer software) | Data extraction and meta-synthesis, p9 |
| 15. Software | State the computer software used, if any | Data extraction and meta-synthesis, p10 |
| 16. Number of reviewers | Identify who was involved in coding and analysis | Data extraction and meta-synthesis, p10 |
| 17. Coding | Describe the process for coding of data (e.g. line by line coding to search for concepts) | Data extraction and meta-synthesis, p10-11 |
| 18. Study comparison | Describe how were comparisons made within and across studies (e.g. subsequent studies were coded into pre-existing concepts, and new concepts were created when deemed necessary) | Data extraction and meta-synthesis, p10-11 |
| 19. Derivation of themes | Explain whether the process of deriving the themes or constructs was inductive or deductive | Data extraction and meta-synthesis, p10-11  Inductive process |
| 20. Quotations | Provide quotations from the primary studies to illustrate themes/constructs, and identify whether the quotations were participant quotations of the author’s interpretation | Meta-synthesis findings (starting on p35)  Quotations and all sources given |
| 21. Synthesis output | Present rich, compelling and useful results that go beyond a summary of the primary studies (e.g. new interpretation, models of evidence, conceptual models, analytical framework, development of a new theory or construct) | Meta-synthesis findings (starting on p35) and discussion (starting on p56) |
