# Supplementary material for: Parents’ experiences of their adolescent child’s depression: a qualitative systematic review and meta-synthesis
Source: BMC Psychol. 2026 Jan 9;14:188. doi: 10.1186/s40359-025-03923-2 (PMC12882447; doi:10.1186/s40359-025-03923-2)
Supplement: Supplementary file 3 — Supplementary Material 3. [file 40359_2025_3923_MOESM3_ESM.docx]

**Medline (Web of Science)**

| S1 | (TI=((parent* or mother or father or caregiver* or guardian* or paternal or maternal or carer*)) OR AB=((parent* or parental or mother or father or caregiver* or guardian* or paternal or maternal or carer*)) OR MH = (Parents)) |
| --- | --- |
| S2 | (TI=((adolescen* or teen* or young person or young people or youth or student*)) OR AB=((adolescen* or teen* or young person or young people or youth or student*)) OR MH = (Adolescent or Students)) |
| S3 | (TI=((depress* or low mood)) OR AB=((depress* or low mood)) OR MH = (Depressive Disorder)) |
| S4 | (TI=((experience* or need* or attitude* or help*seek* or knowledg* or information or qualitative or interview* or focus group*)) OR AB=((experience* or need* or attitude* or help*seek* or knowledg* or interview* or focus group)) OR MH = (Qualitative Research) OR MH = (Help-Seeking Behavior)) |
| S5 | (S1 AND S2 AND S3 AND S4) |

**APA PsycINFO**

| S1 | (TI=((parent* or mother or father or caregiver* or guardian* or paternal or maternal or carer*)) OR AB=((parent* or parental or mother or father or caregiver* or guardian* or paternal or maternal or carer*)) OR MH = (Parents)) |
| --- | --- |
| S2 | (TI=((adolescen* or teen* or young person* or young people* or youth or student*)) OR AB=((adolescen* or teen* or young person or young people or youth or student*)) OR MH = (Adolescent or Students)) |
| S3 | (TI=((depress* or low mood or emotional difficult* or mood)) OR AB=((depress* or low mood or emotional difficult* or mood)) OR MH = (Depressive Disorder)) |
| S4 | (TI=((experience* or need* or attitude* or help*seek* or knowledg* or information or qualitative or interview* or focus group*)) OR AB=((experience* or need* or attitude* or help*seek* or knowledg* or interview* or focus group)) OR MH = (Qualitative Research) OR MH = (Help-Seeking Behavior)) |
| S5 | (S1 AND S2 AND S3 AND S4) |

**EMBASE**

| S1 | 'parent':ti,ab,kw OR 'caregiver':ti,ab,kw OR 'mother':ti,ab,kw OR 'father':ti,ab,kw OR 'paternal':ti,ab,kw OR 'maternal':ti,ab,kw OR 'carer':ti,ab,kw |
| --- | --- |
| S2 | 'adolescent':ti,ab,kw OR 'young people':ti,ab,kw OR 'young person*':ti,ab,kw OR 'teen*':ti,ab,kw OR 'youth':ti,ab,kw |
| S3 | 'depress*':ti,ab,kw OR 'low mood':ti,ab,kw |
| S4 | 'qualitative':ti,kw OR 'experience*':ti,kw OR 'interview*':ti,kw OR 'focus group':ti,kw OR 'mixed method*':ti,kw OR 'need*':ti,kw OR 'view*':ti,kw OR ‘help seek’:ti, kw |
| S5 | (S1 AND S2 AND S3 AND S4) |

**Web of Science Core Collection**

(TI=(parent* or mother or father or caregiver* or guardian* or paternal or maternal or carer*) OR AB=(parent* or parental or mother or father or caregiver* or guardian* or paternal or maternal or carer*)) AND (TI=(adolescen* or teen* or young person or young people or youth or student*) OR AB=(adolescen* or teen* or young person or young people or youth or student*)) AND (TI=(depress* or low mood) OR AB=(depress* or low mood)) AND (TI=(experience* or need* or attitude* or help*seek* or knowledg* or information or qualitative or interview* or focus group*))

**CINAHL**

| S1 | MW parents OR TI parent* OR TI caregiver* OR TI mother* OR TI father* OR TI carer* OR TI maternal OR TI paternal |
| --- | --- |
| S2 | AB parent* OR AB caregiver* OR AB mother* OR AB father* OR AB carer* OR AB maternal OR AB paternal |
| S3 | S1 OR S2 |
| S4 | MW adolescence OR TI adolescent* OR TI teen* OR TI young people OR TI youth OR TI student* |
| S5 | AB adolescent* OR AB teen* OR AB young people OR AB youth OR AB student* |
| S6 | S4 OR S5 |
| S7 | MW depression OR TI depress* OR TI low mood |
| S8 | AB depression* OR AB low mood |
| S9 | S7 OR S8 |
| S10 | MW qualitative studies OR TI qualitative* OR TI experience* OR TI need* OR TI help seeking OR TI knowledge* OR TI interview* OR TI focus group* OR TI attitude* |
| S11 | S3 AND S6 AND S9 AND S10 |
