# Supplementary material for: Parents’ experiences of their adolescent child’s depression: a qualitative systematic review and meta-synthesis
Source: BMC Psychol. 2026 Jan 9;14:188. doi: 10.1186/s40359-025-03923-2 (PMC12882447; doi:10.1186/s40359-025-03923-2)
Supplement: Supplementary file 4 — Supplementary Material 4. [file 40359_2025_3923_MOESM4_ESM.docx]

| **Meta-theme** | **Subtheme** | **Code** | **Code description** |
| --- | --- | --- | --- |
| **Theme 1: Uncertainty with recognizing depression symptoms** | Unawareness of the severity of symptoms | Not realising anything was wrong | Some parents only found out their adolescent was struggling when someone else told them (usually a school professional) |
|  |  | Teenage changes or depression symptoms | Uncertainty over whether the symptoms were signs of typical teenage mood changes, or something more serious like depression |
|  | Noticing changes in adolescent behavior | Noticing adolescent’s withdrawal | Parents started noticing their adolescent was not socialising as much as they used to |
|  |  | Noticing changes but unsure of what they are | Parents noticed their adolescent was acting unusually (e.g. showing signs of irritability and aggression) but were not sure how to interpret them |
| **Theme 2: Understanding the causes of adolescent depression** | Internal causes: guilt, shame and self-blame | Self-blame and shame due to parenting | Feelings of self-blame due to something the parent ‘did wrong’ which led to their adolescent’s depression |
|  |  | Psychoeducation eases the self-blame and guilt | Learning about various factors involved in adolescent depression can ease the self-blame as parents realise parenting alone was unlikely to cause the symptoms |
|  | External causes: pressure and puberty | Role of social interactions | Some parents attributed their adolescent’s symptoms to peer interactions, bullying and pressure from teachers – factors largely beyond their control |
|  |  | Usual teenage behavior | Some parents felt their adolescent’s symptoms were cause by hormonal changes and were expected in the teenage period |
| **Theme 3: Adolescent depression can cause emotional turbulence in parents** | Self-harm and suicidality are distressing for parents | Uncertainty and dread over the prospect | Profound fear over the prospect or anticipation of finding out their adolescent was self-harming or had suicidal ideation |
|  |  | Feelings of shock following self-harm/suicidality | Parents who had already experienced their adolescent self-harming or having suicidal ideation described the profound distress they had gone through |
|  | Heartbreak and worry about the present and future | Heartbreaking to see their adolescent in distress | Parents described the sadness they felt while observing their adolescent’s struggles |
|  |  | Worry about long-term prospects | Parents worried about long-term impacts on their adolescent’s carer and relationships |
| **Theme 4: Adolescent depression affects the whole family** | Changes in parenting | Providing extra parenting | Parents described having to spend more time and use additional efforts to look after their adolescent |
|  |  | Navigating boundaries | Some parents relaxed existing boundaries as to not upset their adolescent; others became stricter in response to the symptoms |
|  | Changes in family dynamics | Changing family planning | Parents described having to change family routines and planning to accommodate the adolescent |
|  |  | Families becoming united | Some families grew closer while supporting the adolescent together |
| **Theme 5: Parents’ experiences with help-seeking** | Parents initiate the help-seeking process | Parents’ curiosity and search for answers | Parents were motivated and driven to learn about adolescent depression and seek help from school and healthcare professionals |
|  |  | Respecting boundaries while help-seeking | Parents described giving their adolescent space and privacy by suggesting a therapist as an external source of support |
|  | Lack of professional support | Not feeling heard | Some parents felt that healthcare professionals dismissed their concerns |
|  |  | Uncertain about the benefits of therapy | Some parents had doubts about the effectiveness of the therapeutic support their adolescent was receiving |
|  | Positive experiences with therapy | Therapy bringing the adolescent back | For some parents, the success of therapy meant their adolescent was back to their ‘usual selves’ |
|  |  | Importance of the therapeutic relationship | Parents placed importance on the individual therapist their adolescent had, as well as their relationship and trust |
|  | Parents’ needs for support themselves | Parents also need support for their own wellbeing | Due to the negative effects on the parents themselves (loneliness, burnout, confusion), parents expressed the need for support for themselves |
|  |  | Parents want to be involved in the support process | There was motivation to get involved through parent-directed resources so that parents can assist in the support process |
| **Theme 6: Stigma and judgment from others** | - | Negative cultural attitudes towards mental health problems | Some parents expressed concerns over mental health related stigma in their communities which they attributed to cultural factors |
|  |  | Fear of judgment from other adults | Parents expressed concern over other adults (friends, parents, teachers, healthcare professionals) judging them for their adolescent’s mental health problems |
